# Uranium Determination in Waters, Wine and Honey by Solid Phase Extraction with New Ion Imprinted Polymer

**DOI:** 10.3390/molecules27175516

**Published:** 2022-08-27

**Authors:** Valentin Georgiev, Ivanka Dakova, Irina Karadjova

**Affiliations:** Faculty of Chemistry and Pharmacy, University of Sofia “St. Kliment Ohridski”, 1164 Sofia, Bulgaria

**Keywords:** ion imprinted polymer, U(VI), 4-(2-Pyridylazo) resorcinol (PAR), surface and ground waters, honey, wine

## Abstract

An analytical method for uranium determination in waters, wine and honey was developed based on solid phase extraction (SPE) with new ion imprinted polymer. The sorbent was synthesized using 4-(2-Pyridylazo)resorcinol (PAR) as a ligand via dispersion polymerization and characterized by SEM for morphology and shape of polymer particles and nitrogen adsorption–desorption studies for their surface area and total pore volume. The kinetic experiments performed showed that the rate limiting step is the complexation between U(VI) ions and chelating ligand PAR incorporated in the polymer matrix. Investigations by Freundlich and Langmuir adsorption isotherm models showed that sorption process occurs as a surface monolayer on homogeneous sites. The high extraction efficiency of synthesized sorbent toward U(VI) allows its application for SPE determination of U(VI) in wine and honey without preliminary sample digestion using ICP-OES as measurement method. The recoveries achieved varied: (i) between 88 to 95% for surface and ground waters, (ii) between 90–96% for 5% aqueous solution of honey, (iii) between 86–93% for different types of wine. The validity and versatility of proposed analytical methods were confirmed by parallel measurement of U in water samples using Alpha spectrometry and U analysis in wine and honey after sample digestion and ICP-MS measurement. The analytical procedure proposed for U determination in surface waters is characterized with low limits of detection/quantification and good reproducibility ensuring its application for routine control in national monitoring of surface waters. The application of proposed method for honey and wine samples analysis provides data for U content in traditional Bulgarian products.

## 1. Introduction

Uranium is an abundant chemical element and occurs as three natural isotopes: ^238^U, ^235^U and ^234^U, all of which are radionuclides with low specific activity. Processes of leaching from granites and other mineral deposits are responsible for the existence of natural uranium in the environment [1,2]. However, the concentrations of natural radionuclides such as uranium in surface waters, and ground waters, are growing last year in connection with closed and remediated uranium mines [3]. The contamination of surface waters and aquifers with radionuclides depends on number of factors: the geology of an area as well as anthropogenic activities such as mining, exploration of minerals, industrial activities, fossil fuel uses, coal ash disposal from thermal power plants, use of phosphate fertilizers, etc. [4]. Due to various processes, the pollution is spreading and uranium is becoming redistributed in all the compartments of the environment, and also in the human food chain [5].

Uranium exposure can induce multifarious health problems as shown by epidemiological and laboratory studies due to its chemotoxicity and radiotoxicity [6] Health risks and toxicological mechanisms are still under investigation and further research is required [7]. It is generally accepted that for soluble and moderately insoluble forms of uranium nephrotoxicity is in the primary consideration—uranium is a chemo-toxic and nephrotoxic chemical element uranium chemotoxicity affects predominantly the kidneys and bones [8]. 

Uranium mobility, toxicity and bioavailability strongly depends on its chemical form [9]. In surface waters U exists in colloidal and particulate form as well as free ions of U^4+^ and U_2_^2+^ and their kinetically labile complexes with inorganic ligands (hydroxides, carbonates, sulphates, phosphates) and inert chelate complexes with humic substances [10]. Uranium background concentrations in European surface water varied in a wide concentration range from less than 0.002 μg/L to 21 μg/L [11]. Uranium is not readily transferred from soil to crops, and also has a low transfer factor from grass to animals. Nevertheless, as a component of the natural environment, uranium is likely to be present as a trace constituent in all foodstuffs. Seeing the potential health hazards of the U, many countries worldwide have adopted the guideline concentration for drinking water quality, mainly based on its chemical toxicity rather than on its radiological toxicity. Due to the high degree of uncertainty in the toxicological data, the proposed value of 30 µg/L is accepted as a provisional in the frame of EU. Concentration limits for food and beverages are almost not available due to the scarce data for U transfer in the food chain and as a general problem due to the lack of analytical methods for U determination at low concentration levels in a complicated matrix. 

Consequently, analytical methods developed for U determination should be in line with U behavior and bioavailability and at the same time should ensure determination of U at a very low concentration levels in various samples. The application of a suitable separation method in such a case is unavoidable and generally solid phase extraction is a preferable procedure due to its simplicity, consumption of low volumes of nontoxic reagents and good repeatability.

Extraction efficiency, selectivity and concentration factors of SPE depend mainly on the sorbent properties which finally resulted in a wide range of sorption approaches developed. Widely used in practice are procedures based on ion exchange or ion sorption on weak and strong ion exchange resins or on chelate resins applied as efficient sorbents for both positively and negatively charged U species [12]. Mostly organic (poly(acrylic acid-co-divinylbenzene), poly(styrene-co-divinylbenzene), polystyrene) or inorganic (amorphous silica gels and mesoporous or nonporous silica) supports are grafted with suitable functional groups for uranium such as amidoxime, dimethyl 2-hydroxy ethyl functional group, murexide, carboxylate modified cyclams [13]. Chelate resins are modified with agents forming highly stable complexes with U: (a) sulfonic acid (SCX); (b) iminodiacetic acid (IDAA); (c) 3,4-hydroxypyridinone (HOPO) [14]. In addition, more efficient support materials such as modified cellulose, activated carbon [15], activated alumina [16], silica/silica gel [17,18,19] have been used again modified with suitable U chelate complex forming reagents. Simplicity and procedures repeatability was increased incorporating magnetic nanoparticles as a core-shell materials for effective sorption of U. Well known is that ion imprinted polymers are sorbents which are characterized with very high selectivity toward imprinted ions. This high selectivity is due to the “key lock” mechanism achieved after incorporation of template species, usually complex of target ion with suitable ligand in the polymer matrix. After elution of the ion the cavity formed is complementary with ion geometry, coordination and size, which results in a very high selectivity [20]. Wide range of ion imprinted polymers (IIP) have been proposed for selective sorption of U mostly in waters [21]. Different types of IIPs were synthesized and applied in analytical procedures for U determination:

- IIPs based on binary and ternary complexes of U. Investigations demonstrated the influence of template composition and porogen on the selectivity of IIP [21,22,23,24,25,26,27,28,29,30,31].
- surface imprinted IIPs on: silica gel, silica particles, silica nanoparticles, amidoxime modified chitosan, modified alginate, cellulose, graphitic carbon nitride composite [32,33,34,35,36].
- IIPs imprinted on magnetic nanoparticles [37,38,39].

Generally, the extraction efficiency and selectivity of IIPs toward U(VI) is mainly based on the chelate complexes incorporated in the polymer matrix. Various ligands have been used including 5,7-dichloroquinoline-8-ol-4-vinylpyridine ternary [20], salicylaldoxime [25], 1-hydroxy-2-(prop-2′-enyl)-9,10-anthraquinone [27], 1-(prop-2-en-1-yl)-4-(pyridin-2-ylmethyl) piperazine [28], benzamidoxime hydrochloride [31]. On the other side the interaction of the ligand used with functional monomers is also important and mostly 4-vinylpyridine, methacrylic acid or 2-hydroxyethyl methacrylate fulfil the requirements. The simplicity of analytical procedure developed depends on the mechanical properties of polymer particles and this leads to the synthesis of surface imprinted polymers using mostly silica gel/chitosan/cellulose as inert supports. The inclusion of magnetic components in the polymer nanoparticles is the easiest way for the separation of sorbent avoiding centrifugation. The properties of the synthesized IIPs are quite different and comparison from the viewpoint of sorbents capacity and detection limits achieved is presented in Table S5. Still the theoretical proposal for the most effective chelate ligand or functional monomer for uranium IIP is not possible and in most cases experimental work is needed for the assessment of extraction efficiency and applicability in analytical procedures.

The aim of the present study is synthesis and characterization of IIP for uranium based on new complexing reagent and further investigations on the extraction efficiency toward U in surface and ground waters as well as in more complex matrices such as wine and honey. The idea is to use newly synthesized IIP for direct quantitative extraction of U from wines (red, rose and white) and monofloral (lime, rapeseed and sunflower) honey without preliminary sample digestion. In this way efficient analytical procedure is developed which allows determination of low levels of U content in waters and in wine and honey—traditional Bulgarian products, using ICP-OES as instrumental method for U measurements in eluates. Analytical figures of merit of the developed method are presented, accuracy is confirmed by parallel analysis of water samples by Alpha spectrometry and for wine and honey after parallel analysis using sample digestion and ICP-MS measurements. The application of the developed procedure ensures data for U content in surface waters, wine and honey samples from different regions of Bulgaria.

## 2. Materials and Methods

### 2.1. Materials

The stock standard solutions of U(VI) were CPA_chem_, solution of Uranium 10.00 g/L in 5% nitric acid (Stara Zagora, Bulgaria). Working standard solutions were daily prepared by appropriate dilution with deionized water (DW) (Millipore Corp., Milford, MA, USA). All reagents were of analytical-reagent grade.

Methacrylic acid (MAA), trimethylolpropane trimethacrylate (TMPTMA), 2,2′-azobisisobutyronitrile (AIBN), 4-(2-Pyridylazo)resorcinol (PAR, as Na salt), uranyl acetate (UO_2_(CH_3_COO)_2_·6H_2_O) (Merck, Darmstadt, Germany), and acetonitrile (ACN) (Labscan, Dublin, Ireland) were used to prepare the U(VI) ion-imprinted and non-imprinted polymer sorbents. Hydrochloric acid (Fisher Chemical™, Waltham, MA, USA) was used for uranium desorption. The pH value of water samples was adjusted with NH_3_ or HNO_3_. 

### 2.2. Apparatus

The concentrations of U were measured by inductively coupled plasma—optical emission spectrometry (ICP-OES, VISTA MPX AXIAL, VARIAN, Mulgrave, Australia). Instrumental parameters were optimized to achieve optimal signal-to-background ratio, according to the Instrument Manual.

The particle’s shape was determined by a scanning electron microscope (SEM, JEOL JSM-5500, Tokyo, Japan). Specific surface area and pore size distribution were measured through nitrogen adsorption–desorption isotherms at 77 K using a Brunauer–Emmett–Teller (BET) analyzer (Quantachrome NOVA 1200e, Quantachrome, Boynton Beach, FL, USA). Elemental analysis was performed using the Euro EA CHNS-O elemental analyzer (EuroVector, Redavalle, Italy). A pH meter (Mettler Toledo; Seven Compact S220-K, Greifensee, Switzerland) was used for pH measurements. In order to separated polymer particles and extracted metal ion, solution in batch experiments, a centrifuge EBA 20 (DJB Labcare Ltd., Newport Pagnell, UK) was used.

### 2.3. Synthesis of U(VI)-IIP and NIIP

**Figure 1:**
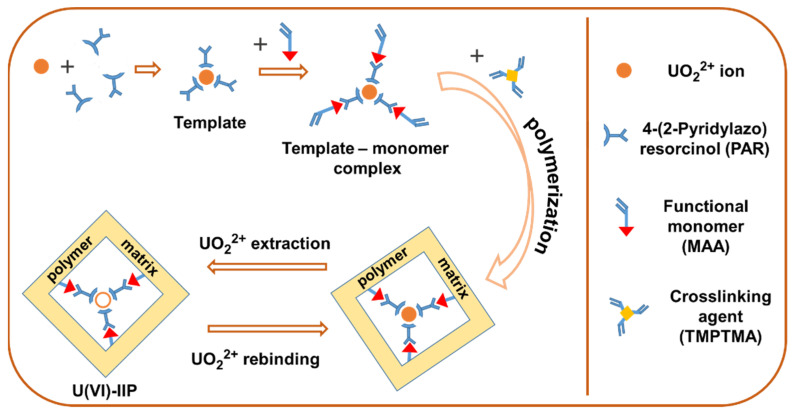
Scheme of the U(VI)-IIP preparation.

The imprinted and non-imprinted polymer gels were synthesized as described earlier with some modifications [40]. In this work, a complex of U(VI) ions with PAR was used as a template and the functional monomer/crosslinking agent/template molar ratio was changed. U(VI)-IIP was prepared via dispersion copolymerization using ACN (25 mL) as a porogen solvent, AIBN (70 mg) as an initiator, MAA (0.58–2.16 mmol) as a functional monomer and TMPTMA (0.96 mmol) as a crosslinking agent in the presence of complexes of the imprinted ion (U(VI)) with PAR as a template species (0.12 mmol). The solution obtained was saturated with dry nitrogen for 15 min and copolymerization was carried out at temperature 333 K for 24 h. Next, polymer particles were recovered by centrifugation and washed with ACN to remove unreacted monomers and other ingredients. Uranium was extracted from the produced polymer networks by several, sequential elution steps using 3 mol/L HCl as eluent. This procedure was repeated until the U concentration (template ions) in the eluate solution was below the LOQ as measured by ICP-OES. Non-imprinted polymer particles (called NIIP) were synthesized in the same way as described above, in the absence of chelating agent and template ion. Finally, the prepared U(VI)-IIP and NIIP particles were dried in a vacuum oven at 333 K. The reaction scheme for U(VI)-IIP preparation is shown in Figure 1.

### 2.4. Static Adsorption/Desorption Experiments of U

A portion of a standard solution containing 20 μg U(VI) was added to a 10 mL deionized water and adjusted to a desired pH value in the range 3–9 by adding HNO_3_ or NH_3_ solution. Polymer gel particles of ca. 100 mg were added to this solution and stirred with an electric shaker for 30 min. The suspension was centrifuged at 5000 rpm. The supernatant solution (effluate) was removed and analyzed by ICP-OES. Next, the polymer particles were washed twice with DW and treated with 5 mL eluent solution (2 mol/L HCl). After centrifugation, the uranium content was measured in the eluate by ICP-OES.

The degree of sorption (*D*_S_, %) of U(VI) ions is calculated by the following equation:

(1) $$D_{S}=\frac{A_{i}\;-A_{\mathrm{eff}}}{A_{i}}\times 100,$$

where *A*_eff_ (µg) is the U(VI) amount in the effluate solution after extraction with polymer gel from a solution with an initial analyte amount *A*_i_ (µg).

The degree of elution (*D*_E_ %) of U(VI) ions retained on the sorbent is defined as:

(2) $$D_{E}=\frac{A_{\mathrm{el}}\;}{A_{i}-A_{\mathrm{eff}}}\times 100,$$

where *A*_el_ (μg) is the amount of uranyl cations in solution after elution process.

### 2.5. Isotherm and Kinetic Studies 

The adsorption capacities of the prepared U(VI)-IIP and NIIP particles were determined by following procedure: 100 mg of adsorbent were mixed with 10 mL of U(VI) solution with increasing initial concentration (2–30 mg/L) under optimum conditions at temperature 298 K. The equilibrium U(VI) concentration after adsorption was measured by ICP-OES. The maximum adsorption capacity of the U(VI)-IIP and NIIP (*Q*_max,exp_) is defined as the amount of the adsorbed U(VI) ions per gram of the copolymer gel and calculated by the following equation:

(3) $$Q_{\max,\exp}=\frac{(C_{0}-C_{e})\cdot V\;}{m\cdot M}$$

where *Q*_max, exp_ is the mass of U(VI) ions adsorbed per unit mass of the sorbent, mg/g; *V* is volume of the solution, L; *m* is the mass of the sorbent, g; *C*_0_ and *C*_e_ are initial and equilibrium concentrations of U(VI) ions in the solution, mg/L; *M* is the atomic mass of uranium, g/mol.

The kinetics of the U(VI) sorption/desorption were investigated in a batch system. A 10 mL of aqueous solution, containing 20 µg U(VI), was treated with 100 mg of polymer gel particles at pH 7 at temperature 298 K for 5–45 min. The contents of U(VI) ions in the effluate and eluate solutions after sorption and elution, respectively, were determined by ICP-OES. 

### 2.6. Interference Studies

A 10 mL aqueous solution containing 20 µg of U(VI) was mixed with solution, containing major cations, characteristic for surface/ground waters, wine and honey (Na, K, Ca, Mg) and anions (carbonate, sulphate, tartarate, mixture of humic acids) at different concentration levels. The mixture was shaken (250 rpm) at pH 7 for 30 min. The retained U(VI) was eluted from the polymer gel particles with 5 mL 2 mol/L HCl. The concentration of U was measured in effluate and eluate solution by ICP-OES.

### 2.7. Analytical Application

#### 2.7.1. Surface/Ground Waters

A 30 mL water sample was transferred in a centrifuge tube and pH adjusted to 7. About 100 mg polymer gel particles were added and mixture shaken for 30 min. The suspension was centrifuged at 5000 rpm and the polymer particles were washed twice with distilled water and treated with 2 mL 2 mol/L HCl. Eluted U was measured by ICP-OES.

#### 2.7.2. Wine Samples

A 20 mL wine (red, rose, white) sample was transferred in a centrifuge tube and pH adjusted to 7. About 100 mg of polymer gel particles were added and the mixture was shaken for 30 min. The suspension was centrifuged at 5000 rpm, polymer particles were washed twice with distilled water and treated with 2 mL 2 mol/L HCl. Eluted U was measured by ICP-OES.

#### 2.7.3. Honey Samples

A 20 mL, 5% honey sample was transferred in a centrifuge tube and pH adjusted to 7. About 100 mg of polymer gel particles were added and the mixture shaken for 30 min. The suspension was centrifuged at 5000 rpm, polymer particles were washed twice with distilled water and treated with 2 mL 2 mol/L HCl. Eluted U was measured by ICP-OES.

## 3. Results and Discussion 

### 3.1. Synthesis and Characterization of U(VI)-IIP and NIIP

The design of ion-imprinted polymers, which ensures high extraction efficiency, requires the correct choice of the chemical components for the polymerization process: chelating agent, functional monomer and crosslinker. The role of the ligand is most important because ion chelation and stability constant of complex formed is directly involved in the recognition process. The choice of molar ratio between monomer and template is the second parameter responsible for the affinity of IIP and determines the accuracy and selectivity of binding sites. In the present study PAR is tested as new ligand for the preparation of ion imprinted polymer with high affinity toward U(VI).

The polymer particles were synthesized by “trapping” technique which includes several stages (Figure 1). The first one was the complex formation between target uranyl ion and specific chelating agent PAR (U(VI)-PAR) in ACN. Next, the template–monomer (prepolymerization) complex was formed by non-covalent interactions between the functional monomer (MAA) and the template molecule (U(VI)-PAR complex). In the next step, the dispersion cross-linking copolymerization of the complexes formed with the TMPTMA as cross-linking agent producing copolymer network. Finally, U(VI) ions were extracted from copolymer gels prepared leaving behind some specific binding sites with functional groups in a predetermined orientation and cavities with special size of templates.

In order to evaluate effectiveness of the synthesis procedure and properties of prepared polymer gels, several IIPs with different compositions were prepared (Table S1). The degree of template molecule incorporation in copolymer network was confirmed by elemental microanalysis of the U(VI)-IIPs. The nitrogen content obtained (between 2.25–3.10 wt.%) suggested that chelating agent PAR was successfully “trapped” into the polymer network of U(VI)-IIPs. The results presented in Table S1 show that increasing the MAA fraction increased the nitrogen content (U(VI)-PAR complex content in polymer matrix, respectively) and the binding capacity of U(VI)-IIPs. This is probably due to the formation of a larger amount of prepolymerization complexes between the MAA and U(VI)-PAR molecules. At the same time, the change in the U(VI)-PAR/MAA/TMPTMA molar ratio does not significantly affect the surface area (*S*_BET_), the total pore volume (*V*_total_) and the average pore diameter (*D*_average_). Finally, the molar ratio 0.12:2.16:0.96 for (U(VI)-PAR:MAA:TMPTMA) was selected as optimal for the synthesis of U(VI)-IIP in the further investigations.

**Figure 2:**
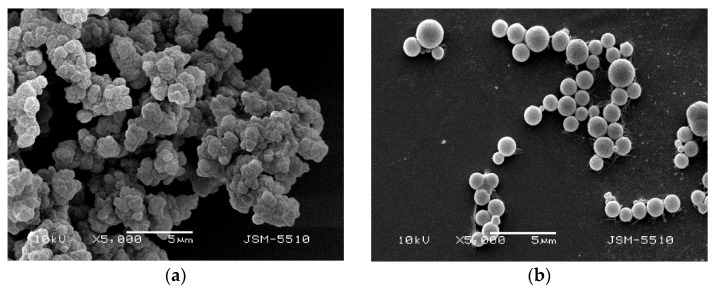
Scanning electron micrograph of prepared copolymer gels at a × 5000 magnification: (**a**) U(VI)–IIP; (**b**) NIIP.

SEM was used to investigate the morphology and shape of U(VI)–IIP and NIIP. As can be seen from Figure 2 only the particles of the non-imprinted polymer gel have close to spherical shape. Their mean diameters, determined from the micrographs, are 1.2 µm. The surface structure and morphology of the U(VI)–IIP is different. Figure 2a shows that the uranyl-imprinted polymer gel is in the form of bigger aggregates of irregular particles with an almost spherical surface, which are packed together.

The results from nitrogen adsorption–desorption isotherms studies showed that the values for the surface area *S*_BET_ (6.5 m^2^/g) and the total pore volume *V*_total_ (0.05 cm^3^/g) of the U(VI)-IIP are lower than these of the non-imprinted polymer gel (27 m^2^/g; 0.10 cm^3^/g). This phenomenon is typical for imprinted copolymer gels and can be explained by the incorporation of U(VI) complex with a chelating agent in the copolymer network, which causes a certain filling of the pores and reduced N_2_ adsorption [41]. The average pore diameter (*D*_average_) values for both imprinted and non-imprinted copolymer gels were 22 nm and 15 nm, respectively, which confirms that they have a mesoporous structure. 

### 3.2. Effect of pH on SPE Efficiency 

**Figure 3:**
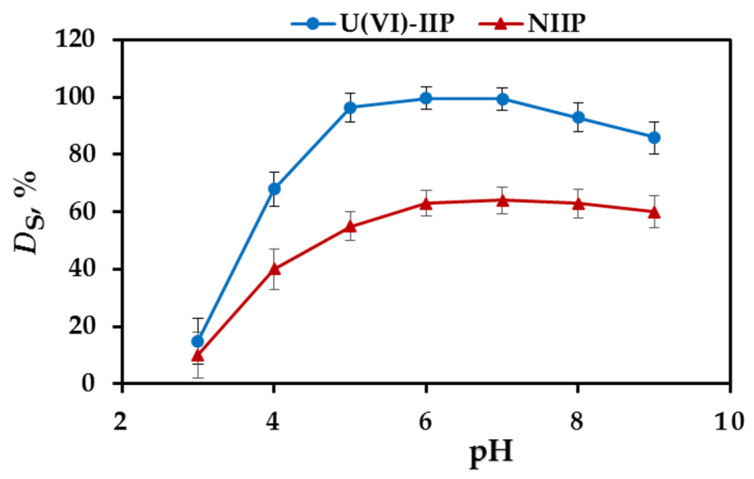
The effect of pH on the extraction efficiency of U(VI) ions with U(VI)-IIP and NIIP (three parallel experiments).

The effect of the sample pH on U(VI) adsorption onto the prepared U(VI)-IIP and NIIP was examined in the pH range 3.0–9.0 and the results are displayed on Figure 3. It is seen that the degree of sorption increased with pH increase, reaching maximum at pH 5–7 and thereafter decreased. These results can be explained by the combined effect of the chemistry of uranyl ions in aqueous solutions and their interactions with both the “trapped” chelating agent and the functional groups present in the polymer network. The changes in adsorption might be related to the pH dependent protonation of chelating ligands and functional monomers in the sorbents. At low pH, the active binding sites in the chelating agents PAR are protonated and positively charged (pK_a1_(PAR) = 3.53 [42] and the adsorption of U(VI) on the sorbent particles is prevented due to the electrostatic repulsion between them. When pH increases, the sorbent surface becomes less positively charged, due to partial deprotonation increasing the ability of the chelating ligands to form complexes with the U(VI) ions. The effect of pH on the degree of protonation of the carboxylic groups in the functional monomer MAA is similar. At pH > 5, the active binding sites in U(VI)-IIP are deprotonated, which ensures the interaction with uranyl ions existing as positively charged species (UO_2_^2+^, [UO_2_(OH)]^+^, [(UO_2_)_2_(OH)_2_]^2+^, [(UO_2_)_3_(OH)_5_]^+^ and [(UO_2_)_4_(OH)_7_]^+^ [43]. These cationic species retained on the U(VI)-IIP particles by complex formation with PAR molecules and by the electrostatic attraction with deprotonated carboxylic group in MAA. At any pH, the sorption affinity of the U(VI)-IIP towards U(VI) ion was higher than that of the NIIP. Quantitative U(VI) sorption (>95%) was achieved in the pH range of 5–7 with U(VI)-IIP, while the extraction efficiency of the NIIP was around 63% for NIIP (Figure 3). The degree of sorption for both U(VI)-IIP and NIIP decreased at pH > 7, most probably related to the partial hydrolysis of uranyl ions forming UO_2_(OH)_2_ and the presence of negatively charged species such as [UO_2_(OH)_3_]^−^, [(UO_2_)_2_(OH)_4_]^2−^ and [(UO_2_)_3_(OH)_7_]^−^ [43]. Finally, the pH of 7 was selected as optimal for the SPE of U(VI) by U(VI)-IIP and NIIP in the further investigations.

### 3.3. Elution Study

The degree of U(VI) ions elution from the loaded U(VI)-IIP particles was investigated by using various concentrations of HCl solutions as desorption agents following the general procedure described in Section 2.4. It was found that 2–3 mol/L HCl is quite effective for quantitative elution (>95%) of U(VI) from the sorbent (Table S2). Thus, 2 mol/L HCl solution was used as an eluent in the subsequent experiments.

The kinetics of the desorption process of U(VI) was investigated by batch procedure with 100 mg of U(VI)-IIP for 5–60 min. Quantitative desorption was reached for 30 min. 

### 3.4. Effect of Contact Time and Adsorption Kinetics

**Figure 4:**
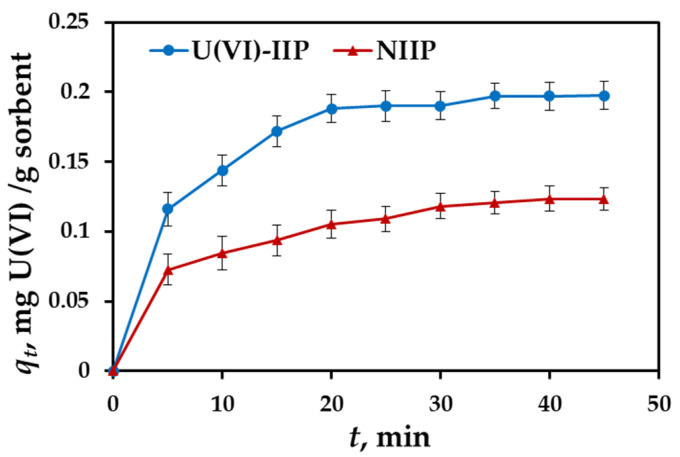
Adsorption kinetics plots of U(VI)-IIP and NIIP towards U(VI) (pH 7; sorbent dose = 100 mg/10 mL; *C*_0_ = 2 mg U(VI)/L, temperature 298 K, three parallel experiments).

The rate of adsorption is one of the important factors for evaluating the sorbent efficiency. In this work, the kinetics experiments were carried out for U(VI)-IIP and NIIP at following conditions: 100 mg/10 mL adsorbent dose, 2 mg/L concentration of U(VI) ions, pH 7 and temperature 298 K. The samples were stirred vigorously for time intervals 5, 10, 15, 20, 25, 30, 35, 40 and 45 min to determine the effect of the contact time on the sorbent binding capacity. It can be seen that the adsorption capacity of U(VI)-IIP towards U(VI) increased rapidly in the first 20 min, then increased at a slower pace and remained unchanged after 30 min (Figure 4). The initial fast adsorption is due to the presence of larger number and more easily accessible specific binding sites on the surface of polymer particles. The results presented in Figure 4 also showed that the adsorption rate of NIIP is slower than that of U(VI)-IIP and its adsorption capacity remains unchanged after 40 min. This can be explained by the lack of imprinted binding sites on the surface of NIIP. 

In order to determine the controlling mechanism of the adsorption process such as mass transfer and chemical reaction, pseudo-first-order (PFO) and pseudo-second-order (PSO) kinetic models were applied to fit the data obtained from adsorption kinetic experiments. PFO model postulated that the rate of occupation of the adsorption sites is proportional to the number of unoccupied sites, while PSO is based on the assumption that the adsorption rate is controlled by the chemical adsorption mechanism [44]. The linear form of equations for these models can be represented as:

pseudo-first-order model:
  (4) $$\ln{(q}_{e}-q_{t})=\ln q_{e}+k_{1}\cdot \;t$$
Pseudo-second-order model:
  (5) $$\frac{t}{q_{t}}=\frac{1}{k_{1}\cdot q_{e}^{2}}+\frac{t}{q_{e}}$$
  where: *q*_e_, *q*_t_—amounts of U(VI) ions retained per mass unit of sorbent at equilibrium and at time *t*, (mg/g), respectively; *k*_1_, *k*_2_—rate constants of pseudo-first-order kinetics model (1/min) and pseudo-second-order kinetics model (g/mg·min), respectively.

The linear plots of pseudo-first-order and pseudo-second-order models for U(VI) ions sorption onto U(VI)-IIP and NIIP are presented in Figure S1a,b (Supplementary Material) and the corresponding kinetics parameters and calculated correlation coefficients are listed in Table 1. Comparison of the results obtained shows that the pseudo-second-order equation appears to be the better-fitting model considering the higher values of the correlation coefficients *R^2^* and the calculated value of *q*_e,calc_, which is closer to the experimental result (*q*_e,exp_). These results prove that the rate limiting step is strong interactions between incorporated in polymer matrix chelating ligand PAR and U(VI) ions. 

The intra particle diffusion model was used to evaluate the role of the diffusion process in the adsorption of U(VI) ions on U(VI)-IIP and NIIP. The linear form of equation for this model can be represented as [45]:

(6) $$q_{t}=k_{\mathrm{diff}\;}\cdot \;t^{1/2}+C$$

where *k*_diff_ is the intra-particle diffusion rate constant (mg/g·min^1/2^) and intercept *C*, obtained by extrapolation of the linear portion of the plot of *q*_t_ versus *t*^1/2^, is an indicator to express the boundary layer thickness.

The plot *q*_t_ versus *t*^1/2^ (Figure S1c) shows that there are two distinct linear parts in the graph with different slopes, which convincingly proves the involvement of more than one step in the adsorption process. The first region could be related to the external mass transfer of the analyte (from bulk solution to the adsorption surface), while the second region could be explained by the internal diffusion of the analyte into the cavities of the polymer gel [46]. The results presented in Table 1 show that for both sorbent materials the calculated *k*_diff_ is higher for the first adsorption step than for the second step. This proves that the first step occurs at a higher adsorption rate. The boundary layer thickness values (*C*) are different from zero, indicating that the adsorption of U(VI) ions on the polymer gels is achieved by surface adsorption, which is controlled by the mass transfer resistance in the external liquid film and by pore diffusion [47].

### 3.5. Effect of Initial U(VI) Concentration and Adsorption Isotherms

**Figure 5:**
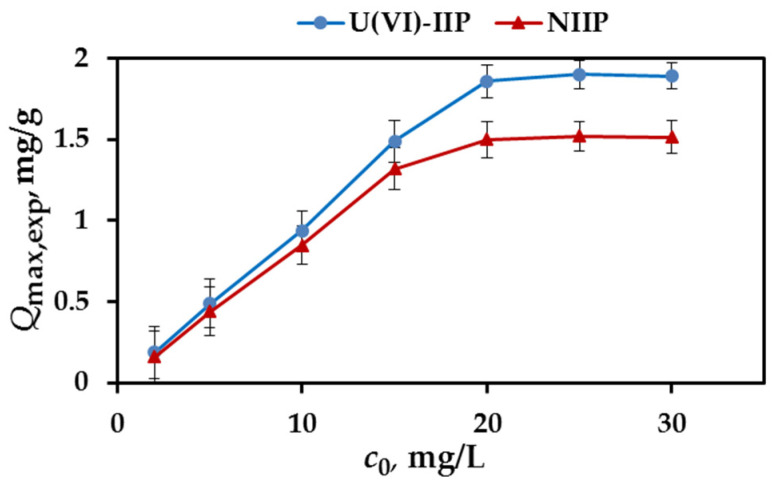
Effect of initial concentration of U(VI) on adsorption capacity of U(VI)-IIP and NIIP (pH 7; contact time = 30 min; temperature 298 K, three parallel experiments).

In order to evaluate the effect of initial U(VI) concentration on the adsorption capacity of U(VI)-IIP and NIIP, batch experiments were conducted according to the procedure described in Section 2.5. Adsorption isotherms constructed with the experimental data showed that the amount of adsorbed U(VI) per unit mass of the sorbent increased with the initial concentration of U(VI), and reached plateau values, determining the maximal experimental adsorption capacity, *Q*_max,exp_ (Figure 5). The value of *Q*_max,exp_ of U(VI)-IIP is higher than that of NIIP—1.89 mg/g vs. 1.35 mg/g (Table 2). These results confirm that the cavities created after removal of the template U(VI) ions from the polymer network ensures higher affinity of U(VI) ions to the imprinted than to the non-imprinted polymer gels. 

Freundlich and Langmuir adsorption isotherm models were used to describe the relationship between equilibrium concentration and adsorption capacity during the adsorption process. The applicability of the isotherm models was studied by judging the correlation coefficients, *R*^2^ values. The Freundlich isotherm model can be applied to multilayer adsorption, with non-uniform distribution of adsorption heat and affinities over the heterogeneous surface [48]. The linearized Freundlich equation is expressed by Equation (7) as follows:

(7) $$\ln Q_{e}=\ln k_{F}+n^{-1}\cdot \ln C_{e}$$

where *C*_e_ (mg/L) is the equilibrium concentration of U(VI) in the solution, *Q*_e_ (mg/g) is the adsorption capacity of the adsorbed U(VI) ions onto the sorbents at equilibrium, *k_F_* and *n* are Freundlich constants incorporating all factors that affect the adsorption process such as capacity and intensity.

The Langmuir isotherm theory assumes that the sorption process occurs in a surface monolayer of homogenous sites which number is fixed [48]. It can be expressed in linear form as Equation (8):

(8) $$\frac{C_{e}}{Q_{e}}=\frac{C_{e}}{Q_{\max}}+\frac{1}{b\cdot Q_{\max}}$$

where *Q*_max_ (mg/g) is the theoretical maximum adsorption capacity, *b* (L/mg) is the Langmuir constant.

The obtained results are presented graphically in Figure S2 and the parameters of each model are shown in Table 2. Analyzing the data presented in Table 2, the correlation coefficients obtained for Langmuir isotherm (*R*^2^: 0.9986 and 0.9997 for U(VI)-IIP and NIIP, respectively) have higher values compared with the values obtained when experimental data are modeled using Freundlich isotherm (*R*^2^: 0.9128, 0.8507 for U(VI)-IIP and NIIP, respectively). This might be accepted as a proof that sorption process occurs as a surface monolayer on a homogeneous sites. The theoretical adsorption capacities *Q*_max,teor_ agreed very well with experimentally obtained values, thus confirming the validity of assumptions for adsorption in monomolecular layer.

To predict the favorability of an adsorption system, the essential characteristics of the Langmuir equation can be expressed in term of a dimensionless factor, *R*_L_, which was defined as [48]:

(9) $$R_{L}=\frac{1\;}{1+b\cdot C_{0}}$$

According to the literature, the isotherm is irreversible, favorable, linear or unfavorable if *R*_L_ = 0, 0 < *R*_L_ < 1, *R*_L_ = 1 or *R*_L_ greater than 1, respectively [48]. As seen in Table 2, the *R*_L_ values are in the range of 0 < *R*_L_ <1 indicating that the adsorption of U(VI) ions on U(VI)-IIP and NIIP is favorable.

### 3.6. Interference Studies 

Potential interferences from major cations and anions in waters/wine/honey (known to form complexes with U(VI)) were studied at different relevant concentration levels. The results obtained (Table 3) showed high recoveries in the range 85–99% depending on the concentration and type of ion. The most serious interferent is HCO_3_^−^, most probably due to the competitive complex formation. Finally, in order to test the combined action of potential interferents recovery experiments in real mineral waters were carried out. As seen from Table 4, for highly mineralized waters a standard addition method should be used for calibration. 

### 3.7. Analytical Application

To test the potential of synthesized U(VI)-IIP for U determination, different types of surface water samples (river, lake, Black Sea) were collected, filtered through a cellulose membrane filter (0.22 μm pore size, Millipore, Burlington, MA, USA) and spiked with U at different concentration levels. Each experiment was performed in triplicate and for each sample new sorbent was used. The results presented in Table 5 showed that recovery depends on water mineralization, mainly on the levels of HCO_3_^−^ and typically varied between 88 and 95%. In the same time procedure developed is characterized with good repeatability, RSD for all recovery values is between 3–8%. Although recovery values below 90% are acceptable a standard addition method has to be proposed for U quantification in mineral waters with high mineralization. The recoveries achieved for surface waters showed that synthesized U(VI)-IIP allows quantitative determination of U in various types of waters and might be applied in monitoring programs. 

The experiments were carried for potential application of IIP for U determination in wine without preliminary digestion. Wine samples (red, rose and white) were spiked with known amount of U and pass through the proposed analytical procedure (see Section 2.7). The results obtained are presented in Table 6 and demonstrate that maximal sample volume is 20 mL with recoveries achieved above 92%. 

The potential application of IIP for U determination in honey is tested for 5% aqueous solutions of honey obtained after simple dissolution of 5 g honey in 100 mL distilled water. The results achieved, presented in Table 7 showed that 5% aqueous solution of honey ensures quantitative recoveries if 20 mL sample is used. 

As seen the whole analytical procedure is very simple and might be performed in one vessel thus minimizing eventual loses or analyte and possible contaminations (Figure S3). In addition, high extraction efficiency of synthesized IIP eliminates the necessity for preliminary digestion step for wine and honey samples.

### 3.8. Analytical Figures of Merit

An analytical procedure was developed for U determination in surface/ground waters, wine and honey based on sorption on U(VI)-IIP, see Section 2.7. Limit of detection/quantification (LOD/LOQ), for U, defined as three/ten times the standard deviation of the blank signal (optimal sorbent amount 100 mg, eluted with 2 mL 2 mol/L HCl) using ICP-OES as instrumental method are: 0.05/0.15 μg/L for surface/ground waters, 0.07/0.2 μg/L for wines and 1.0/3.0 μg/kg for honey. As can be seen even by using less expensive method such as ICP-OES almost background values for U might be determined in waters and really low levels of U content in wine and honey might be reached. The calibration graphs were linear from the LOQ to 30 μg/L (maximum concentration assayed) for waters and wine and from LOQ to 50 μg/kg for honey. The relative standard deviations varied in the range between 5 to 9% for waters, 5–11% for wines and 6–11% for honey.

The validity of results for U content in waters obtained by the proposed analytical method was checked by parallel analysis using Alpha spectrometry. In order to compare results achieved by spectrometry they were recalculated in Bq/L. Very good agreement achieved between parallel results as presented in Table S3 confirmed the versality and applicability of analytical method for U determination based on newly synthesized U(VI)-IIP. As far as data for valence state of U in wine and honey is not known the reliability of data for U content might be confirmed only after complete sample digestion and measurement by more sensitive instrumental method. That is why the validity of results for wine and honey were confirmed by parallel analysis using sample digestion and ICP-MS. Very good agreement between results achieved (see Table S4) verified the applicability of proposed method for U determination in wine and honey without preliminary sample digestion and by using cheaper measurement method as ICP-OES. 

To test the reusability of the U(VI)-IIP, the sorbent was applied for several adsorption/desorption cycles using 2 mol/L HCl for elution. The results obtained indicated that IIP for U might be used for at least 20 adsorption/desorption cycles without significant (less than 10%) change of adsorption capacity and extraction efficiency. 

The repeatability of the synthesis procedure was also checked by using IIPs obtained from different batches for parallel determination of U in water, wine and honey samples. Statistically unsignificant differences were found between analytical results obtained for U content. Most probably optimal reagents content as well as simplicity of synthesis procedure ensures this high repeatably. 

The analytical applicability of synthesized IIP for U was compared with already published literature data as shown in (Table S5). As seen the adsorption capacity of proposed U(VI)-IIP is better than the one, reported by Tavengwa et al. [49,50] and a bit lower than those reported by Gladis et al. [22], Singh et al. [25], Fasihi et al. [27], Zhang et al. [29] and Qian et al. [39]. Due to the low uranium concentrations in studied samples, this capacity value is considered high enough for practical work in analytical laboratories. In order to support this conclusion more than 100 river and stream water samples from nonpolluted and polluted (regions in the vicinity of abandon uranium mines) were analyzed using developed SPE analytical procedure. The results for nonpolluted waters varied between 0.01 and 5 µg/L while for polluted streams, concentrations as high as 20–400 µg/L were found. The U content in about 100 wine samples from different winery regions in Bulgaria were determined using developed procedure. Relatively low U concentrations in the range between <LOQ to 3.5 µg/L were found for studied wine samples with median value of 0.224 µg/L. The proposed analytical procedure was also used for the analysis of monofloral (lime, rapeseed, sunflower) honey samples from different regions in Bulgaria. The results for U content varied between 1.23 µg/kg to 12.32 µg/kg. Statistical comparison of results obtained showed that significantly higher values for U concentrations were found in lime honey, independently of region. It might be suggested that botanical origin is more important to define U content in honey than geographical region. Applicability results undoubtedly showed that relatively low adsorption capacity of U(VI)-IIP did not affect its ability to retain U from even in complex matrices, indicating that U(VI)-IIP could be a promising sorbent for selective determination of U in surface/ground waters, wine and honey.

Additionally, quantification limits and reproducibility of developed analytical method ensures efficient determination of U in traditional Bulgarian products. 

## 4. Conclusions 

An analytical method is proposed for U determination in waters, wine and honey based on SPE with ion imprinted polymer synthesized via dispersion copolymerization using new ligand 4-(2-Pyridylazo)resorcinol as a template. The high extraction efficiency toward U achieved under optimal conditions: pH 7 for sorption and 2 mol/L HCl for elution, allows quantitative recoveries for U in highly mineralized waters and in wine and honey samples without preliminary digestion. Analytical procedure developed for U determination ensures low detection/quantification limits using ICP-OES for U measurements and good reproducibility, relative standard deviations varied between 5 to 9% for waters, 5–11% for wines and 6–11% for honey. Accuracy of developed method was confirmed through comparative analysis for waters by alpha spectrometry and for wine and honey by parallel analysis of digested wine and honey samples and ICP-MS measurements. Quantification limits achieved and reproducibility satisfy the requirements of national monitoring programs for U control in surface waters. Application of developed method ensures data for U content in wine and honey traditional products from Bulgaria. 

## Figures and Tables

**Table 1:** Comparison of pseudo-first-order kinetics and pseudo-second-order kinetics constants and experimental and calculated *q*_e_ values. (pH 7; sorbent dose: 100 mg/10 mL; *C*_0_ = 2 mg U(VI)/L, temperature 298 K).

Model	Parameters	U(VI)-IIP	NIIP
Pseudo-first-order model	*q*_e,exp_ (mg/g)	0.19	0.13
	*q*_e,calc_ (mg/g)	4.20	11.00
	*k*_1_ (1/min)	0.151	0.072
	*R* ^2^	0.945	0.974
Pseudo-second-order model	*q*_e,calc_ (mg/g)	0.21	0.14
	*k*_2_ (g/mg.min)	1.078	1.108
	*R* ^2^	0.998	0.996
Intra-particle diffusion model Region 1	*k*_diff_ (mg/g min^1/2^)	0.033	0.014
	C (mg/g)	0.042	0.040
	*R* ^2^	0.996	0.990
Intra-particle diffusion model Region 2	*k*_diff_ (mg/g min^1/2^)	0.005	0.008
	C (mg/g)	0.164	0.072
	*R* ^2^	0.790	0.844

**Table 2:** Experimental adsorption capacities, Langmuir and Freundlich isotherm parameters obtained by linear fitting for the U(VI)-IIP and NIIP at temperature 298 K.

Polymer Gel	*Q*_max,exp_ mg/g	Langmuir Isotherm Model				Freundlich Isotherm Model		
		*Q*_max,teor_*,* mg/g	*b,* L/mg	*R^2^*	*R*_L_	*k*_F_	*n*	*R^2^*
U(VI)-IIP	1.89	1.91	1,80	0.9986	0.02–0.22	20.25	2.35	0.9128
NIIP	1.35	1.37	4.85	0.9997	0.01–0.10	1.83	3. 42	0.8507

**Table 3:** Recoveries for U determination in the presence of different concentrations of major cations and anions in waters (three parallel determinations).

Interferent	Recovery, % [Mean ± SD] at Concentration:			
	10 mg/L	50 mg/L	100 mg/L	200 mg/L
HCO_3_^−^	98 ± 2	95 ± 3	90 ± 3	85 ± 4
SO_4_^2−^	>99	>99	91 ± 3	93 ± 3
Cl^−^	>99	>99	>99	98 ± 2
Na^+^	>99	>99	98 ± 2	96 ± 3
K^+^	>99	>99	97 ± 2	97 ± 2
Ca^2+^	>99	>99	97 ± 3	95 ± 3
Mg^2+^	>99	>99	96 ± 3	95 ± 2
tartrate	>99	>99	93 ± 3	92 ± 4
Humic substances, 2 mg/L	98 ± 2			

**Table 4:** Recoveries for U determination in mineral waters with different compositions (three parallel determinations).

Mineral Water Sample	HCO_3_^−^, mg/L	CO_3_^2−^, mg/L	SO_4_^2−^, mg/L	Cl^−^, mg/L	Recovery, % [Mean ± SD]
Gorna Bania	17	22	22	9	95 ± 2
Bankia	62	12	51	10	94 ± 2
Devin	89	21	28	11	91 ± 3
Bachkovo	92	18	31	7	90 ± 2
Hisar	120	15	21	9	88 ± 3

**Table 5:** Recoveries for U determination in different types of surface waters (three parallel determinations).

Water Sample	Recovery, % Mean	RSD, %
River Iskar	95	4
River Maritsa	92	5
Lake Ogosta	91	5
Black sea water (Burgas gulf)	93	2
Tap water Sofia	94	3

**Table 6:** Recoveries for U determination in wine samples (three parallel determinations).

Wine Sample	Recovery, % Mean ± SD		
	10 mL Sample	20 mL Sample	30 mL Sample
Red (merlot)	97 ± 3	92 ± 3	80 ± 5
Rose	98 ± 2	94 ± 3	82 ± 5
White (Muskat)	98 ± 2	93 ± 3	81 ± 5

**Table 7:** Recoveries for U determination in honey samples, pH = 7 (three parallel determinations).

Honey Sample (5% Aqueous Solution)	Recovery, %, Mean ± SD		
	10 mL Sample	20 mL Sample	30 mL Sample
Honey (lime)	>99	96 ± 2	84 ± 4
Honey (rapeseed)	>99	97 ± 2	86 ± 4
Honey (sunflower)	>99	96 ± 2	85 ± 5

## Data Availability

Not applicable.

## Supplementary Materials

The following supporting information can be downloaded at: https://www.mdpi.com/article/10.3390/molecules27175516/s1, Figure S1: Kinetic analysis of U(VI) adsorption on U(VI)-IIP and NIIP: the linear fitting curves of pseudo-first-order reaction (a) and pseudo-second-order reaction (b) and intra-particle diffusion model (c). (pH 7; sorbent dose = 100 mg/10 mL; *C*_0_ = 2 mg U(VI)/L, temperature 298 K); Figure S2: Langmuir (a) and Freundlich (b) isotherms for adsorption of U(VI) on the U(VI)-IIP and NIIP; Figure S3: Scheme of analytical procedure for U determination in surface waters using U(VI)-IIP; Table S1: Polymerization conditions for the preparation of copolymer gels (70 mg AIBN; 25 mL ACN; TMPTMA (0.96 mmol); U(VI)-PAR (0.12 mmol); T = 60 °C; 24 h) and nitrogen content, surface area (*S*_BET_), total pore volume (*V*total), average pore diameter (*D*_average_) and adsorption capacities of U(VI)-IIPs; Table S2: Degree of elution (*D*_E_, %) for U(VI) from U(VI)-IIP using different eluents (10 mL); Table S3: Parallel analysis of tap water by the proposed analytical method and direct Alfa spectrometry; Table S4: Comparative analysis by the proposed method and standard method based on microwave digestion of wine/honey sample and ICP-MS measurement; Table S5: Comparison of different U(VI) ion imprinted polymer sorbents.

## Sample Availability

Samples of the compounds (U(VI) ion imprinted and non-imprinted polymers) are available from the authors.
