# Supplementary material for: Uranium Determination in Waters, Wine and Honey by Solid Phase Extraction with New Ion Imprinted Polymer
Source: Molecules. 2022 Aug 27;27(17):5516. doi: 10.3390/molecules27175516 (PMC9457621; doi:10.3390/molecules27175516)
Supplement: Supplementary file 1 [file molecules-27-05516-s001.zip › molecules-1837725-supplementary.pdf]

molecules

MDPI

Supplementary Materials

Uranium Determination in Waters, Wine and Honey by Solid Phase Extraction with New Ion Imprinted Polymer

Valentin Georgiev, Ivanka Dakova * and Irina Karadjova

Faculty of Chemistry and Pharmacy, University of Sofia "St. Kliment Ohridski", 1, J. Bourchier Blvd., 1164 Sofia, Bulgaria

* Correspondence: i.dakova@chem.uni-sofia.bg

Figures

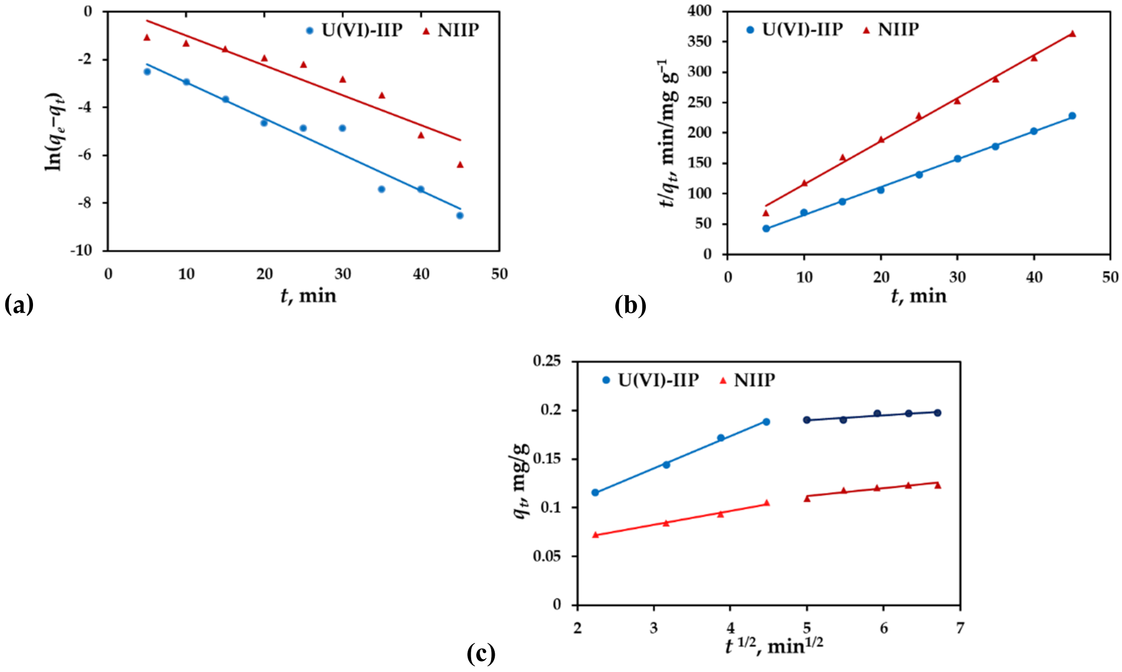

Figure S1. Kinetic analysis of U(VI) adsorption on U(VI)-IIP and NIIP: the linear fitting curves of pseudo-first-order reaction (**a**) and pseudo-second-order reaction (**b**) and intra-particle diffusion model (**c**). (pH 7; sorbent dose = 100 mg/10 mL; C_0 = 2 mg U(VI)/L, temperature 298 K).

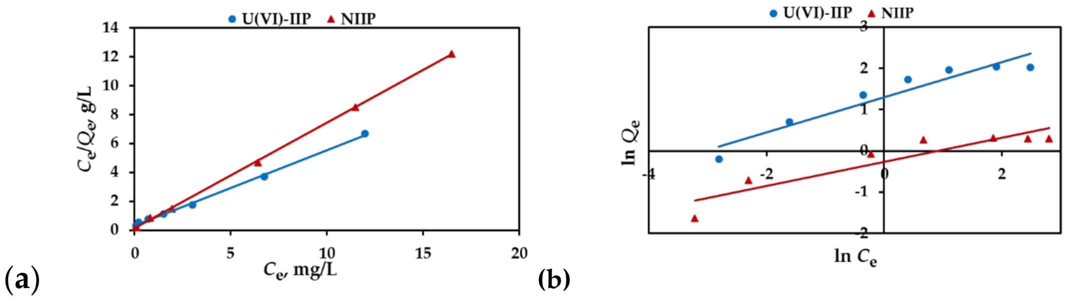

Figure S2. Langmuir (**a**) and Freundlich (**b**) isotherms for adsorption of U(VI) on the U(VI)-IIP and NIIP.

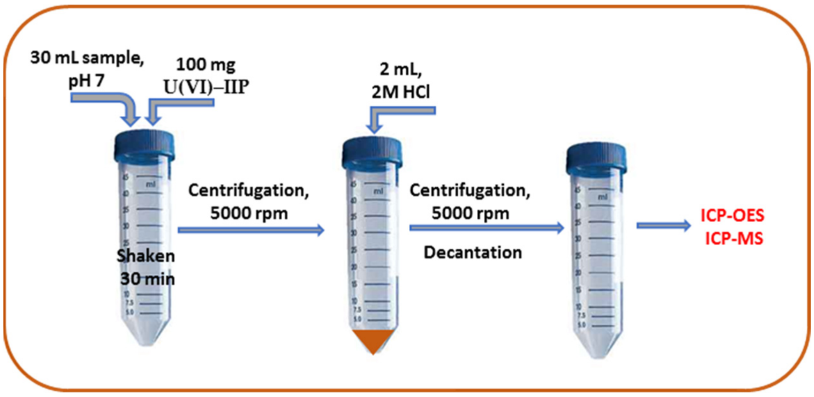

Figure S3. Scheme of analytical procedure for U determination in surface waters using U(VI)-IIP.

Tables

Table S1. Polymerization conditions for the preparation of copolymer gels (70 mg AIBN; 25 mL ACN; TMPTMA (0.96 mmol); U(VI)-PAR (0.12 mmol); T = 60 °C; 24 h) and nitrogen content, surface area (S_{BET}), total pore volume (V_{total}), average pore diameter ($D_{average}$) and adsorption capacities of U(VI)-IIPs.

Copolymer gel	MAA (mmol)	N content (% wt.)	S_{BET} (m^2/g)	V_{total} (cm^3/g)	$D_{average}$ (nm)	Capacity (mg/g)
U(VI)-PAR-1	0.58	2.25	7.0	0.04	24	0.96
U(VI)-PAR-2	1.16	2.63	6.2	0.06	20	1.29
U(VI)-PAR-3	1.80	2.85	6.4	0.02	20	1.55
U(VI)-PAR-4	2.16	3.10	6.5	0.05	22	1.89
U(VI)-PAR-5	2.50	2.96	6.5	0.04	24	1.80

Table S2. Degree of elution (D_E, %) for U(VI) from U(VI)-IIP using different eluents (10 mL).

Desorption agent	Concentration	D_E, %
HCl	0.5 mol/L	48±4
	1 mol/L	83±3
	2 mol/L	96±2
	3 mol/L	99±2

Table S3. Parallel analysis of tap water by the proposed analytical method and direct Alfa spectrometry.

U content, Bq/L	Tap water 1		Tap water 2	
	Proposed analytical method*	Alpha spectrometry	Proposed analytical method	Alpha spectrometry
^{234}U	0.29±0.02	0.27±0.03	0.48±0.03	0.50±0.04
^{238}U	0.28±0.03	0.27±0.02	0.47±0.04	0.46±0.03

*—in this case ICP-MS was used as instrumental method.

Table S4. Comparative analysis by the proposed method and standard method based on microwave digestion of wine/honey sample and ICP-MS measurement.

Sample	U content	
	Proposed analytical method, µg/L	ICP-MS after digestion, µg/L
Red wine (Cabernet)	0.74±0.04	0.77±0.02
White wine (Muskat)	0.43±0.03	0.41±0.02
Honey (sunflower)	1.43±0.12	1.51±0.08
Honey (lime)	3.52±0.21	3.58±0.09

Table S5. Comparison of different U(VI) ion imprinted polymer sorbents.

Chelating agent/Matrix	Capacity, mg/g	Instrumental method	LOQ, µg/L	Ref.
2,4-dioxopentan-3-yl methacrylate – ethylene glycol dimethacrylate	15.3	ICP-OES		[29]
oleic acid coated magnetic particles	1.06	ICP-OES		[50]
1-hydroxy-2-(prop-2′-enyl)-9,10-anthraquinone – ethylene glycol dimethacrylate	12.4	UV-VIS		[27]
Salicylaldoxime / magnetic IIP	1.2	ICP-OES	-	[49]
Salicylaldoxime / ɣ-methacryloxypropyltrimethoxysilane modified magnetic nanoparticles – MAA – ethylene glycol dimethacrylate	5.4	ICP-OES		[38]
Salicylaldoxime / 4-vinylpyridine – MAA – ethylene glycol dimethacrylate	15.3	UV-VIS	5	[25]
5,7-dichloroquinoline-8-ol / 4-vinylpyridine – styrene – divinyl benzene	34	UV-VIS	2	[22]
N-hydroxyethylacrylamide and 1-vinylimidazole@ magnetic microspheres	146	ICP-OES	0.8 mg/L	[39]
IIP-grafted silica	-	ICP-OES	0.09 (LOD)	[34]
4-(2-Pyridylazo)resorcinol / MAA – TMPTMA	1.89	ICP-OES<br>ICP-MS	0.15<br>0.003	This work
